# Applying the Cognitive Model of Post-Traumatic Stress to Examine the Role of Appraisals, Trauma Memory, and Coping Strategies Following Pediatric Injury: A Systematic Review

**DOI:** 10.1007/s40653-025-00695-0

**Published:** 2025-02-19

**Authors:** Jamie Patronick, Kelly R. Molloy, Sabrina J. Bothwell, Shari L. Wade

**Affiliations:** 1 Department of Psychology, University of Cincinnati, Cincinnati, OH USA; 2 Division of Rehabilitation Medicine, Cincinnati Children’s Hospital Medical Center, Cincinnati, OH USA; 3 Department of Pediatrics, University of Cincinnati College of Medicine, Cincinnati, OH USA

**Keywords:** Pediatric injury, Medical trauma, Post-traumatic stress, Cognitive model

## Abstract

Following a traumatic injury, 25–57% of children will develop clinically significant post-traumatic stress symptoms (PTSS). The cognitive model of post-traumatic stress disorder posits that PTSS develop based on the encoding of trauma memories and the individual’s appraisals of the event and its sequelae. In addition, the behaviors and cognitive strategies used to cope with distress are proposed to serve as causal and maintaining factors for PTSS. The purpose of this study was to systematically review and summarize the role of psychological processing factors, including cognitive appraisals, trauma memory, and coping styles, on the development of PTSS in children following injury. Systematic searches were conducted in May 2024 for articles published at any time in PubMed, APA PsycInfo, and PTSDpubs databases. Included articles analyzed the association between cognitive appraisals, trauma memories, and/or coping strategies and PTSS in children (ages 5–18) who received medical treatment for a single-incident traumatic injury. Data were extracted related to study and sample characteristics, measures, and findings regarding the association between psychological processes and PTSS. The quality of included studies was assessed using the Quality Assessment Tool for Observational Cohort and Cross-Sectional Studies. The titles and abstracts of 836 articles were screened, and 105 full text articles were reviewed, resulting in 34 eligible articles. Articles were rated as being of “good” (44%) or “fair” (56%) quality. Studies examined cognitive appraisals (*n* = 18), trauma memory characteristics (*n* = 15), and coping strategies (*n* = 13). Several factors were associated with PTSS, including maladaptive cognitive appraisals, guilt, avoidant coping, and decreased cohesion, accuracy, and context in the trauma narrative. This review provides support for the cognitive model of PTSS when applied to pediatric injury populations. Limitations of the current literature are noted, including lack of evidence of temporal associations and relationships between variables in the cognitive model. Findings can be used to identify cognitive-behavioral treatment targets and ascertain the ideal timing for screening and preventative interventions.

**Supplementary Information:**

The online version contains supplementary material available at 10.1007/s40653-025-00695-0.

Traumatic injuries are the leading cause of death and disability in children and youth in the United States (Centers for Disease Control and Prevention, 2021). The most common mechanisms of injury in childhood include falls, being struck by or against an object, lacerations, motor vehicle accidents, and recreational activities (Owens et al., 2008). Improvements in intensive care and surgical intervention, as well as the advent of trauma systems within the past several decades, have shifted the focus of the trauma system from survival to maximizing quality of life following injury (Namachivayam et al., 2010).

Despite improvements in medical treatment and physical health outcomes following traumatic injury, youth face many psychological and social stressors during and following discharge from the hospital. Youth are at particular risk for depression (Kilpatrick et al., 2003), anxiety (Gold et al., 2008), and post-traumatic stress symptoms (PTSS) related to their injury, medical treatment, and recovery. Traumatic injuries are associated with a peri-trauma period, which involves the accident itself, transport to the hospital, invasive medical procedures, and communication of diagnoses or injury effects. Following the peri-trauma period, there is an early, ongoing, and evolving response, which includes active medical treatment, hospitalization, and medical care demands. Lastly, longer-term or chronic PTSS can extend past the end of active medical treatment (Price et al., 2016).

As many as 25–57% of children develop clinically significant PTSS following an injury (Kahana et al., 2006; Schreier et al., 2005). PTSS include intrusive memories, nightmares and sleep disturbances, negative emotionality, avoidance of trauma memories, hyperarousal, and difficulties with focus. PTSS within the first month of experiencing a traumatic event is diagnosed in the DSM-5 as acute stress disorder (ASD; American Psychiatric Association, 2022). A diagnosis of post-traumatic stress disorder (PTSD) for children and adolescents over age six applies when PTSS persist after one-month post-trauma. Symptoms must include at least one intrusion symptom (e.g., intrusive memories), avoidance of memories and/or external reminders of the event, at least two negative alterations in cognitions and mood (e.g., persistent negative emotional state), and at least two alterations in arousal (e.g., hypervigilance; American Psychiatric Association, 2022). For children ages six and under, there is a lower threshold for symptoms and a recognition of the ways that symptoms may manifest differently (e.g., arousal symptoms may manifest as severe temper tantrums; Scheeringa et al., 2003).

There are several challenges in applying the diagnostic criteria of PTSD to children. In particular, several diagnostic criteria (e.g., sleep disturbances, decreased concentration) overlap with other mood and anxiety disorders, raising some concerns for a lack of sensitivity of the diagnosis in children (Cohen & Scheeringa, 2009). Further, there are developmental considerations when assessing for PTSD in children, primarily due to the largely internalizing nature of DSM-5 PTSD symptoms. Children may have difficulty verbalizing symptoms such as intrusive memories or negative alterations in mood, leading trauma symptoms to be underrecognized or overshadowed by behavioral symptoms, such as temper tantrums, separation anxiety, and somatic complaints (Cohen & Scheeringa, 2009). Child and parent agreement regarding PTSS is also generally poor (Scheeringa et al., 2006). Additionally, although avoidance symptoms are a hallmark diagnostic criterion, it can be difficult to tease apart in an interview whether a child is avoiding thoughts or discussion of their trauma or if they are coping appropriately with the traumatic experience (Cohen & Scheeringa, 2009). Thus, it is important for clinicians assessing children following a potentially traumatic event to use developmentally appropriate measures, include caregivers’ perspectives on observable behaviors and avoidance symptoms, and conduct a thorough interview that includes an understanding of the temporal relationship between the event and symptom onset.

PTSS are associated with lasting psychological impacts, including lower health-related quality of life (Holbrook et al., 2005; Landolt et al., 2009) and poorer adaptive functioning (Zatzick et al., 2008). In addition, chronic stress hyperarousal leads to poorer physical health outcomes due to chronic dysregulation of stress systems (Kendall-Tackett, 2009). Importantly, these negative downstream consequences can occur regardless of whether the child meets full diagnostic criteria for PTSD. Because PTSS typically develop and begin to impact functioning after hospital discharge, they are often unrecognized and untreated by the trauma system.

Research has demonstrated that there are several common trajectories of PTSS throughout the post-injury period. While most children (57%) are resilient to stress following injury and PTSS remain consistently low, some children (33%) exhibit PTSS immediately post-trauma but recover quickly, and as many as 10% of children will go on to have chronic stress symptoms (Le Brocque et al., 2009). Meta-analyses have identified several demographic and pre-injury risk factors for the development of PTSS in children. Female gender, lower pre-injury cognitive skills, previous traumatic life events, lower family socioeconomic status, and experiencing the loss of a loved one during the accident are associated with higher PTSS, with small-to-medium effect sizes (Trickey et al., 2012). Low social support and poorer family functioning have a medium-to-large effect size in predicting PTSS (Trickey et al., 2012). There is some evidence suggesting young children are at greater risk for acute stress but not persistent PTSS than older children (Brosbe et al., 2011). Increased ability to understand and recall the traumatic medical event also appears to be a risk factor for the development of PTSS in children with chronic illness (Barakat et al., 2006).

More research is needed to identify modifiable predictors of these PTSS trajectories, which can inform interventions to support children in the acute period of trauma and prevent the development of chronic PTSS. One conceptual model originally developed in the adult literature is the cognitive model of PTSD, developed by Ehlers and Clark (2000). The model proposes that chronic PTSS occurs if individuals process the traumatic event and/or its sequelae in a way that produces a sense of current threat. A sense of current threat is caused by (1) individual differences in the appraisal of the trauma, and (2) individual differences in the nature of the memory of the event and its link to autobiographical memories (Ehlers & Clark, 2000). Once the memory of the traumatic event is activated, so is the perception of current threat, leading to PTSS. A cascade of behavioral and cognitive responses and coping behaviors follows these symptoms in order to decrease perceived threat in the short-term (e.g., avoidance of triggers). However, these behaviors maintain or worsen symptoms over time (Ehlers & Clark, 2000). There is an additional biological component to this cycle, given that initial fear responses (e.g., increased heart rate) following a trauma exposure are thought to contribute to fear conditioning, which results in further hyperarousal (Nixon et al., 2010).

For children in medical settings, objective indicators of injury or disease severity are generally poor predictors of the development of psychological distress (Trickey et al., 2012), indicating the importance of subjective factors related to a child’s experience with a traumatic medical event. However, as the cognitive model of PTSD was developed for adults, few studies investigate its applicability to children and adolescents. There are similarities and key differences in how children and adults appraise and encode memories that may be relevant to the applicability of the cognitive model of PTSD. At a young age, children utilize sensory information to process their surroundings and generate memories and appraisals of their environment; however, as they grow older and develop linguistic skills, children acquire a greater capacity to consolidate information and encode memories (Ruetti et al., 2019). Specifically, children’s autobiographical memory develops starting in infancy and throughout childhood, allowing for encoding and recall of events occurring within their own lives (Schneider & Ornstein, 2015). By 18 to 24 months of age, young children can encode, store, and later retrieve memory representations associated with past events they have experienced (Ross et al., 2019). Across preschool and school years, children’s autobiographical memory performance continues to improve with the further development of language, increased comprehension of the event, and an increased understanding of the self (Bauer & Larkina, 2019). Importantly, the cognitive and emotional appraisals of an event (e.g., the interpretation, emotional valence, and meaning attributed to an event) influence the attention and encoding processes that occur during an emotional experience. For example, in both children and adults, events that lead to negative emotions and appraisals of threat are often associated with effortful processing and an evaluation of possible obstacles to achieve goals, which may support the encoding of memories for the event (Stein, 2002).

Research supports the influence of child cognitive appraisals following trauma on the development of PTSD, with a large effect size (Mitchell et al., 2017). In particular, negative appraisals about the self (e.g., “I will never be the same again”) and the world (e.g., “the world is a scary place where I am highly vulnerable”) appear to be associated with increased PTSS (Mitchell et al., 2017). Emerging research has highlighted how adaptive or functional post-traumatic appraisals may protect against the development of PTSS. For example, among injured adults and children following natural disasters or war, perceived ability to function after traumatic event (i.e., coping self-efficacy) is inversely related to PTSS (Bosmans et al., 2015; Langley & Jones, 2005). Further, adaptive appraisals (e.g., “I have put the event in the past”, “other people think I am brave”) are inversely related with maladaptive appraisals and PTSS in children (Hitchcock et al., 2015; de Haan et al., 2021).

Using autobiographical memory mechanisms, aspects of peri-trauma memory processing also lead to PTSS following the event, with more superficial or “data-driven” processing of memories (e.g., memories of the physical or sensory aspects of the trauma) leading to more symptoms than contextual processing of the meaningful aspects of the trauma (McKinnon et al., 2008). Overgeneral memory refers to the impaired retrieval of specific, single-incident events from autobiographical memory (e.g., when asked to retrieve a specific memory, the child reports a category of event instead), and may also be associated with PTSS (Hitchcock et al., 2014a, b). Lastly, certain avoidant or emotion-focused coping strategies, such as distraction, social withdrawal, and poor emotional regulation are associated with increased distress and are more common in children who meet criteria for PTSD (Stallard et al., 2001).

Despite this support for specific cognitive processes in predicting PTSS in children, and some similarities in memory for negative emotional events across the lifespan (Stein, 2002), there are other potential factors that may contribute to conceptualizing a cognitive model of PTSD for youth, particularly following injury. Children have less autonomy related to their environments than adults, which may impact the relationship between coping responses and PTSS. As youth rely on caregivers to appraise danger and develop a narrative of the event, factors such as caregiver distress, PTSS, coping styles, and parent-child communication about the injury also play a role in the development and maintenance of child PTSS (Wise & Delahanty, 2017). Lastly, following injury, children may also be coping with physical limitations, decreased social participation, and poorer quality of life, which may have a bidirectional influence on PTSS (Schnurr et al., 2006).

Unlike other risk factors for PTSS, cognitive processing factors may be modifiable by intervention. Trauma-focused cognitive behavioral therapy (TF-CBT; Cohen et al., 2016) teaches strategies for re-appraisal of maladaptive cognitions, formation of a trauma narrative, gradual exposure techniques, and practice with adaptive coping strategies. TF-CBT has been shown to reduce maladaptive appraisals in children following sexual assault (McLean et al., 2015), motor vehicle accidents, interpersonal violence, and witnessed violence (Smith et al., 2007). In particular, the effects of TF-CBT on PTSS, depressive symptoms, and general mental health symptoms appear to be mediated by changes in post-traumatic appraisals (Jensen et al., 2018). Evaluating the literature on psychological processes following injury and medical treatment will contribute to our understanding of the development of PTSS in this population, inform early identification of risk factors for PTSS, and improve intervention at the individual level and prevention at the trauma system-level.

The objective of this review was to evaluate the influence of psychological processing variables (cognitive appraisals, trauma memory, and coping styles) on child PTSS following injury. The review was limited to single-incident traumatic injuries. Theories of pediatric medical traumatic stress highlight commonalities in features of traumatic stress reactions following injury and chronic illness (Price et al., 2016). However, there are also unique aspects associated with a traumatic injury that are different from chronic stressors such as prolonged illness or long-term trauma exposures such as abuse. For example, traumatic injuries are associated with exposure to the emergency medical system (e.g., medical transport, emergent procedures), and there is typically little time to prepare the child for what to expect. Further, following an injury related to an accident, children may have additional stressors related to witnessing the injuries or deaths of others. Thus, combining illness and injury populations may mask some of the potential subtle differences in patient and family experiences (Kahana et al., 2006). To cast a wide net in evaluating the state of the literature on psychological processing factors, and because many studies include injuries as a result of both unintentional/accidental and intentional injuries (e.g., assault), we chose not to exclude articles based on the intentionality of the injury if it was a single-incident trauma.

## Methods

### Search Strategy

The search took place between May 22 and June 1, 2024, and used PubMed (searched May 22, 2024), PTSDPubs (searched May 22, 2024), and PsychInfo (searched June 1, 2024). Within each database, the following keywords were entered and searched within titles and abstracts:

(pediatric injury OR accident* OR traumatic injury OR pediatric emergency OR pediatric intensive care unit OR PICU OR ICU OR unintentional injury) AND (“child” OR “children” OR “youth” OR “young people” OR “adolescent” OR “adolescence” OR “childhood” OR “teen” OR “teenager” OR school age) AND (PTSD OR post-traumatic stress disorder OR posttraumatic stress disorder OR “complex PTSD” OR acute stress OR “acute stress disorder” OR “medical trauma” OR “post-traumatic stress symptoms” OR “posttraumatic stress symptoms”) AND ((“memory” OR “trauma memory”) OR (appraisal* OR “maladaptive appraisal” OR cognitive process*) OR (“cognitive bias” OR “interpretive bias” OR “attribution” OR post-traumatic cognition* OR posttraumatic cognition*) OR (“coping behavior” OR “coping behaviour” OR “coping strategies” OR “coping skills” OR “coping responses” OR “coping style” OR “coping” OR “avoidance” OR “thought suppression” OR “distraction” OR “safety behaviors” OR “safety behaviours” OR “selective attention” OR “attentional bias” OR “rumination” OR “anxiety”)).

Reference lists from included studies were also searched manually for relevant articles, and these articles were located using Google Scholar. Articles were not filtered by year of publication, as this is a relatively new area of research. In accordance with systematic review best practices, no other filters or limits were used within included databases (Lefebvre et al., 2019).

### Eligibility Criteria

Covidence, a systematic review software, was used for the article screening and data extraction process. After automated duplication removal, titles and abstracts of articles were reviewed for eligibility by one reviewer. If they met initial eligibility criteria, the full text was then reviewed. Articles were included in the review if they assessed the influence of a psychological processing variable on PTSS in children (ages 5–18) who received medical treatment (emergency department, inpatient, or outpatient) for a single-incident traumatic injury. Because subthreshold PTSS is still associated with functional impairment as well as comorbidities such as depression and suicidal ideation (Marshall et al., 2001), studies were not required to only include children meeting diagnostic criteria for PTSD. Articles including young children (ages 0–5) were excluded due to differences in the manifestation of trauma symptoms and the emerging cognitive-linguistic abilities that may influence their processing of trauma (De Young et al., 2021). This study chose to define “psychological processes” as any variable that fit within the three primary constructs from the cognitive model of PTSS as outlined by Ehlers and Clark (2000):

1. Trauma memory: a measure of the elaboration and integration of the memory for the traumatic event, due to incomplete processing of the event. This construct includes data-driven processing, lack of self-referent processing, and peri-trauma dissociation.
2. Appraisals: how an individual makes meaning from or attributes the traumatic event and/or its sequelae.
3. Coping strategies: behavioral and cognitive strategies to control perceptions for threat and trauma triggers. Behaviors may be functional (e.g., support-seeking) or dysfunctional (e.g., distraction). Cognitive strategies may be adaptive (e.g., regulation) or maladaptive (e.g., rumination).

Inclusion criteria included: (a) English-language articles, (b) empirical articles with at least partly quantitative analyses, (c) publication in a peer-reviewed journal, and (d) articles that examined either child self-report and/or caregiver proxy-reports of child psychological processing and PTSS. Articles were excluded if they: (a) reported the impact of psychological processing on another outcome (e.g., depression), (b) examined psychological processing after other types of traumatic events (e.g., natural disasters, witnessed trauma), (c) reported the results of an intervention study (unless only pre-intervention data were used in analyses), (d) only reported parent outcomes, or (e) included children following moderate or severe traumatic brain injury, given the potential confounding factors of neurocognitive deficits and post-traumatic amnesia in this population (Greenspan et al., 2006).

### Data Extraction and Synthesis

To summarize article characteristics and findings and ensure systematic coding, the primary author developed a data extraction form. One reviewer independently completed data extraction for the full texts. Once the final list of articles was identified, the following data were collected from each reference: (1) sample characteristics (age, sex, race/ethnicity, injury characteristics), (2) recruitment setting, (3) data collection time points, (4) sample size, (5) types of psychological processing variables measured (categorized into trauma memory, appraisals, and/or coping strategies), (6) measures of cognitive variables and PTSS, and (7) a summary of findings related to the review’s aims.

Study characteristics and demographics were tabulated. Study findings were grouped and tabulated based on the type of psychological processing variable and are described narratively below. Effects of psychological processing variables on PTSS were considered significant based on statistical significance values (*p* < .05), and effect sizes were reported with results when available.

### Quality Assessment

**Fig. 1:**
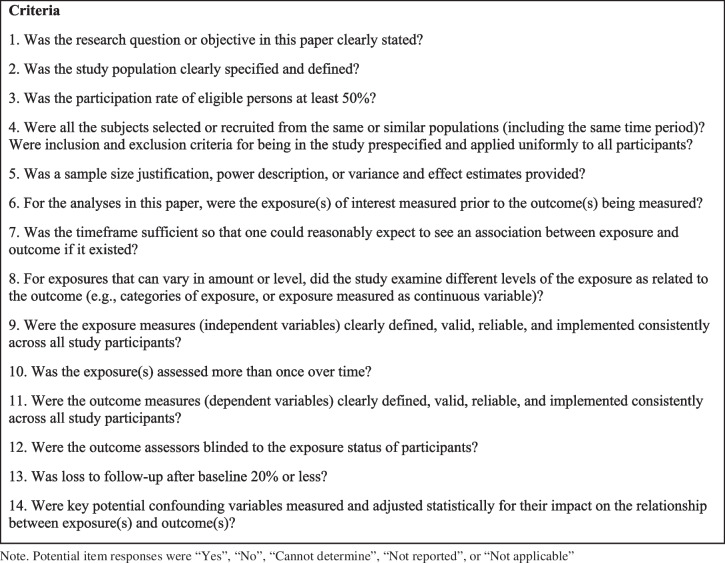
NHBLI quality assessment tool for observational cohort and cross-sectional studies (NHLBI, 2019)

Two reviewers independently assessed study quality using the Quality Assessment Tool for Observational Cohort and Cross-Sectional Studies, designed by the NIH National Heart, Lung, and Blood Institute (NHLBI; NHLBI, 2019). The tool was designed for use when reviewing the quality of evidence in a literature review and focuses on rating concepts that are key for critically appraising the internal validity of a study. It includes 14 items that evaluate potential flaws in study methodology or implementation, including sources of bias (e.g., patient selection and attrition), confounding, and study power (see Fig. 1 for the full tool used). For each item, the reviewers selected “yes” or “no” based on the evidence provided in the article. Per publisher guidelines, responses were not tallied to create an overall quality score (NHLBI, 2019). Instead, each “no” response was considered as a potential source of bias, and each reviewer appraised an overall summary judgement of “good”, “fair”, or “poor” by considering the likelihood that the “no” response caused doubt in the results of the study. Reviewers met periodically to resolve discrepancies and reach consensus for final ratings. A third author aided in resolving discrepancies. Interrater reliability and confidence intervals were calculated using Krippendorff’s alpha (Hayes & Krippendorff, 2007).

## Results

**Fig. 2:**
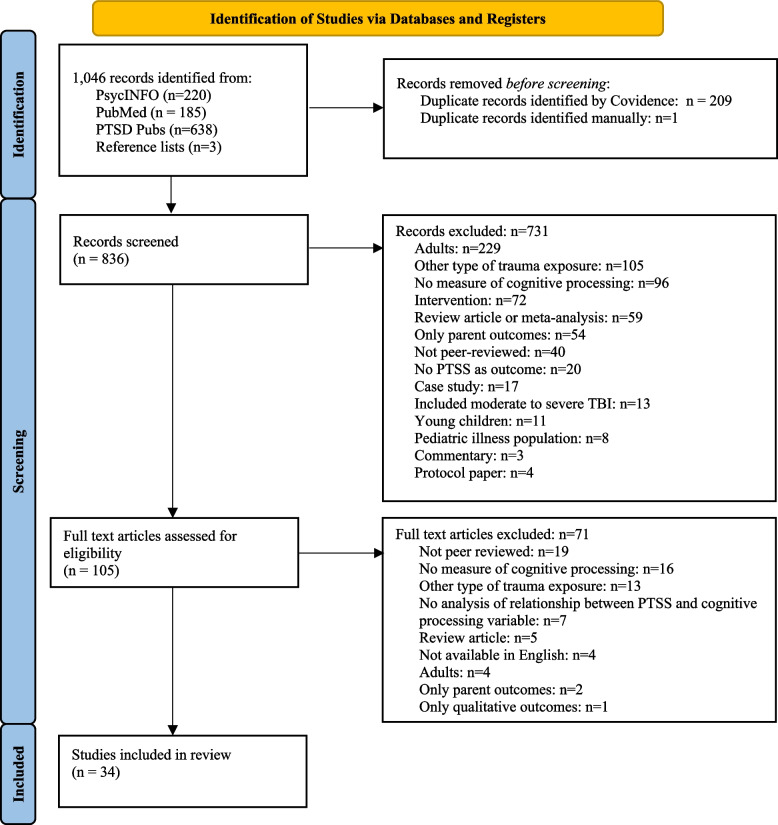
PRISMA flow diagram for the systematic review

The initial search across databases yielded a total of 1,046 records, which was narrowed to 836 publications after removing duplicates. After the title and abstract review, 105 records were retrieved to review the full text. A full text review resulted in 34 articles to be included (see Fig. 2 for PRISMA diagram). Primary reasons for article exclusion were that the study investigated other types of trauma exposure (e.g., child maltreatment, natural disasters), or the study participants were out of the age range (i.e., young children or adults). Studies were also frequently excluded if they did not specifically examine the relationship between a psychological processing variable and PTSS, or if another outcome was used (e.g., depressive symptoms).

### Quality Assessment Ratings

A summary of quality assessment rankings for the 34 articles reviewed is presented in Table 1. Fifteen articles (44%) were assigned a rating of “good”, indicating few concerns that “no” responses on the Quality Assessment Tool led to significant bias in results. Ratings of “fair” were assigned to 56% (*n =* 19) of articles, indicating a potential for biased results or that “no” responses had an unclear effect on the quality of results. No articles were rated as poor, indicating that no studies were judged to have concerns in multiple domains that substantially lowered the author’s confidence in the study’s findings. The most frequent concern for study quality was related to a lack of sample size, power justification, and/or effect size estimate (item 5; “No” selected for 82% of studies). Only one study reported effect sizes (Bray et al., 2018). Additionally, 53% (*n* = 18) of studies were rated as not having a clearly specified and defined study population (item 2). Per NIHLBI guidelines, cross-sectional studies are rated as “no” automatically for items 6 (exposures measured prior to the outcomes) and 7 (timeframe sufficient to see an association between exposure and outcome). Therefore, 44.1% (*n* = 15) of articles were rated as “no” on these criteria. Interrater reliability by item varied from poor to perfect agreement. The average Krippendorff’s alpha coefficient was 0.479.

**Table 1:** Quality assessment ratings

Authors	Research question	Population	Participation rate	Recruitment	Sample size and power	Exposures before outcome	Timing	Levels of exposure	IVs	Assessed over time	DVs	Blinding	Loss to FU	Confounds	Final quality rating
Meiser-Stedman et al., 2007b	Yes	No	No	Yes	No	Yes	Yes	Yes	Yes	Yes	Yes	N/A	No	No	Fair
McKinnon et al., 2017a	Yes	No	NR	Yes	No	Yes	Yes	Yes	Yes	Yes	Yes	NR	Yes	Yes	Good
Hitchcock et al., 2015	Yes	No	Yes	Yes	No	Yes	Yes	Yes	No	Yes	Yes	No	Yes	No	Fair
Marsac et al., 2014	Yes	Yes	CD	Yes	No	No	Yes	No	Yes	Yes	Yes	N/A	No	No	Fair
Aaron et al., 1999	Yes	Yes	Yes	Yes	Yes	No	No	Yes	Yes	No	Yes	Yes	N/A	No	Good
Stallard, 2003	Yes	No	No	Yes	No	No	No	Yes	No	No	Yes	NR	N/A	Yes	Fair
Saxe et al., 2005	Yes	No	Yes	Yes	No	Yes	Yes	Yes	Yes	Yes	Yes	NR	Yes	Yes	Good
Kenardy et al., 2007	Yes	No	CD	Yes	No	Yes	Yes	No	No	Yes	Yes	NR	Yes	No	Fair
O’Kearney et al., 2007	Yes	No	CD	Yes	No	No	No	Yes	No	No	Yes	NR	N/A	Yes	Fair
McKinnon et al., 2008	Yes	Yes	Yes	Yes	No	No	No	Yes	Yes	No	Yes	N/A	N/A	Yes	Good
Ellis et al., 2009	Yes	No	Yes	Yes	No	No	No	Yes	Yes	No	Yes	N/A	N/A	Yes	Good
Nixon et al., 2010	Yes	Yes	Yes	Yes	No	Yes	Yes	Yes	Yes	Yes	Yes	N/A	NR	Yes	Good
Vincken et al., 2012	Yes	No	NR	Yes	Yes	Yes	Yes	Yes	Yes	Yes	Yes	N/A	No	No	Good
Marsac et al., 2013	Yes	Yes	CD	No	No	Yes	Yes	Yes	Yes	Yes	Yes	N/A	Yes	No	Fair
Morris et al., 2013	Yes	Yes	CD	Yes	No	No	No	Yes	Yes	No	Yes	N/A	N/A	Yes	Fair
Hitchcock et al., 2014a	Yes	Yes	CD	Yes	Yes	Yes	Yes	Yes	Yes	Yes	Yes	NR	Yes	Yes	Good
McGuire et al., 2021	Yes	No	No	Yes	Yes	Yes	Yes	Yes	No	Yes	Yes	Yes	Yes	Yes	Fair
McKinnon et al., 2017b	Yes	Yes	CD	Yes	No	Yes	Yes	Yes	Yes	Yes	Yes	NRs	NR	Yes	Fair
Meiser-Stedman, 2009	Yes	Yes	No	Yes	No	Yes	Yes	Yes	No	Yes	Yes	N/A	No	Yes	Good
de Haan et al., 2019	Yes	No	NR	Yes	No	No	No	Yes	Yes	No	Yes	N/A	N/A	Yes	Fair
Meiser-Stedman et al., 2007a	Yes	Yes	No	Yes	No	No	No	Yes	No	No	No	Yes	N/A	Yes	Fair
Stallard et al., 2001	Yes	No	No	Yes	No	Yes	Yes	No	Yes	Yes	Yes	NR	Yes	Yes	Fair
Seasons & Morrongiello, 2024	Yes	Yes	CD	Yes	No	No	No	Yes	No	No	No	N/A	N/A	No	Fair
Stallard & Smith, 2007	Yes	No	CD	Yes	No	No	No	Yes	No	No	Yes	NR	N/A	Yes	Fair
Bryant et al., 2007	Yes	No	No	Yes	No	Yes	Yes	Yes	Yes	Yes	Yes	Yes	Yes	Yes	Good
Salmond et al., 2011	Yes	No	No	Yes	Yes	No	No	Yes	Yes	No	Yes	Yes	N/A	Yes	Good
Salmon et al., 2007	Yes	No	No	Yes	No	No	No	Yes	Yes	No	Yes	N/A	N/A	Yes	Fair
Bray et al., 2018	Yes	Yes	CD	Yes	Yes	Yes	Yes	Yes	No	Yes	Yes	NR	Yes	Yes	Good
Ehlers et al., 2003	Yes	No	Yes	Yes	No	Yes	Yes	Yes	No	Yes	No	NR	Yes	Yes	Fair
Marsac et al., 2017	Yes	Yes	Yes	Yes	No	Yes	Yes	Yes	Yes	Yes	Yes	N/A	No	Yes	Good
Marsac et al., 2016a	Yes	Yes	CD	No	No	Yes	Yes	No	Yes	Yes	No	No	CD	Yes	Good
de Haan et al., 2021	Yes	No	No	Yes	No	No	No	Yes	Yes	No	Yes	N/A	N/A	No	Fair
Haag et al., 2015	Yes	Yes	Yes	Yes	No	No	No	Yes	No	No	Yes	N/A	N/A	Yes	Fair
Hildenbrand et al., 2020	Yes	Yes	No	Yes	No	Yes	Yes	Yes	Yes	Yes	Yes	N/A	No	Yes	Good

*FU *Follow-up,* N/A* not applicable, *NR *not reported, *CD* cannot determine

### Study Characteristics

The sample demographics, recruitment information, and study design for each article are summarized in Supplemental Table 1. In total, the 34 studies represented 3,515 children, with sample sizes ranging from 40 to 688. Studies were published between 1999 and 2024 and were conducted in Australia (*n* = 12), United Kingdom (*n* = 9), United States (*n* = 8), Switzerland (*n* = 3), the Netherlands (*n* = 1), and Canada (*n* = 1). The age of participants ranged from five to 17. Most samples (*n* = 30, 88.2%) were majority male. Of the 16 studies (47.1%) that reported the racial/ethnic identities of their sample, 13 (81.3%) had a predominantly White sample. Injury mechanisms varied and included assaults, road traffic accidents, falls, burns, and bicycle or other recreational vehicle accidents. Some studies exclusively examined one type of injury, such as road traffic accidents (e.g., Stallard & Smith, 2007) or skateboarding injuries (Seasons & Morrongiello, 2024). Other studies included all children treated in a trauma center for any type of single-incident trauma. Participants were recruited from a variety of settings, including only in the emergency department (*n* = 12, 35.3%), following admission to the ICU or regular ward (*n* = 14, 41.2%), emergency room or inpatient units (*n* = 4, 11.8%), or following any medical treatment, including in inpatient or outpatient settings (*n* = 2, 5.9%). One study (Seasons & Morrongiello, 2024) recruited children retrospectively using flyers and online postings and required self-report of an injury requiring medical treatment within the past year.

Fourteen studies (41.2%) were cross-sectional and assessed psychological processing variables and their relationship with PTSS between several hours post-injury (Morris et al., 2013) to up to one year post-injury (Seasons & Morrongiello, 2024). The majority (*n* = 20, 58.8%) of the studies were longitudinal, with all studies first assessing cognitive processing and PTSS within one month of the injury. Follow-up data collection for longitudinal studies took place between six weeks and eight months post-injury. No studies examined symptoms past eight months post-injury.

### Measurement of PTSS

For the majority of studies (*n* = 28, 82.4%), PTSS as a continuous outcome was measured using questionnaires. One study combined three samples for analysis and used a binary outcome (presence or absence of clinically significant PTSS; Marsac et al., 2016a, b). The most commonly used questionnaires were the Child PTSD Symptom Scale (CPSS; *n* = 10; Foa et al., 2001), the Impact of Event Scale- Revised (RIES-C; *n* = 7; Perrin et al., 2005), the UCLA PTSD Reaction Index (RI; *n* = 5; Steinberg et al., 2013), and the Acute Stress Checklist for Children (ASC-Kids; *n* = 3; Kassam-Adams, 2006). The remaining studies (*n* = 5, 14.7%) used a clinician-administered diagnostic interview to determine whether the child met DSM-IV or DSM-5 criteria for ASD or PTSD and used a binary outcome (meeting or not meeting criteria for PTSD or ASD).

The majority (*n* = 32, 94.1%) of studies used child self-reported symptoms as the outcome measure. One study used parent-proxy report of symptoms (Kenardy et al., 2007). One study (Ehlers et al., 2003) used parent-proxy report to verify certain symptoms (e.g., play reenactment) for younger children during a diagnostic interview.

Twenty-two studies reported the number of children who met criteria for clinically-significant PTSS or an ASD or PTSD diagnosis. Rates ranged from 3% (Haag et al., 2015) to 38% (Salmond et al., 2011).

### Psychological Processes

#### Cognitive Appraisals

Supplemental Table 2 contains a summary of the measures used and key findings for the 18 studies that examined the role of cognitive appraisals.

##### Measurement

All studies that measured cognitive appraisals used either child self-report questionnaires (*n =* 17) or child interviews (*n* = 1) to assess appraisals. No studies used a parent-proxy measure of child appraisals. The most common measure was the Child Post-Traumatic Cognitions Inventory (CPTCI; Meiser-Stedman et al., 2009a, b), used by 10 studies. The CPTCI measures negative or dysfunctional post-traumatic cognitions about oneself and the world within two subscales (“Fragile Person in a Scary World” and “Permanent and Disturbing Change”). Two studies (Marsac et al., 2017; Hildenbrand et al., 2020) used a set of items on the ASC-Kids that relate to appraisals of the injury event. Two studies measured adaptive or functional post-traumatic appraisals using the Adaptive Appraisals Questionnaire (AAQ; *n* = 1, Hitchcock et al., 2015) or the Functional Posttraumatic Cognitions Questionnaire (FPTCQ; *n* = 1; de Haan et al., 2021). The remaining studies used a variety of non-validated Likert scale items to assess specific types of appraisals and their relationships to PTSS. For example, Haag and colleagues (2015) examined guilt appraisals using one Likert scale item (i.e., “Do you feel guilty for causing the accident?”). Seasons and Morrongiello (2024) examined appraisals of vulnerability prior to the accident and attributions of the accident being related to “bad luck.” Stallard (2001) examined appraisals retrospectively from child interview questions designed to assess PTSS (i.e., child perceptions of the effects of the injury and their emotional and physical recovery).

##### Findings 

Overall, 17 out of 18 studies (94.4%) found that negative or maladaptive cognitive appraisals were significantly positively related to PTSS or predicted meeting criteria for ASD or PTSD. Overall, negative appraisals were found to account for 13–44% of the variance in PTSS across studies. Appraisals of threat during the event were positively related to acute PTSS, and the relationship between appraisals and PTSS was mediated by escape coping (Marsac et al., 2016a). Feeling guilty for causing the accident was found to predict PTSS severity as well as intrusion and avoidance symptoms, but not hyperarousal symptoms (Haag et al., 2015). Similarly, anger towards oneself was significantly correlated with PTSS, although anger towards others was not (Meiser-Stedman et al., 2007a). In contrast, Seasons and Morrongiello (2024) found no significant effects of injury-related appraisals or attributions of bad luck on PTSS.

When examining the specific subscales of the CPTCI, Meiser-Stedman (2009) found that scores on the Permanent and Disturbing Change subscale mediated the relationship between acute and chronic PTSS. The effect was not significant for the Fragile Person in a Scary World subscale. In contrast, Bryant and colleagues (2007) found that only the Fragile Person subscale significantly predicted chronic PTSS, and this subscale was the strongest predictor of PTSS in another study (Salmon et al., 2007). In terms of specific relationships between appraisals and PTSS, one study found that negative appraisals mediated the relationship between child perceived social support and PTSS (Hitchcock et al., 2015), while Ellis and colleagues (2009) failed to find a similar relationship. deHaan and colleagues (2019) found that appraisals mediated the relationship between younger age and lower parental educational level on child PTSS.

Functional or adaptive appraisals were found to be negatively correlated with PTSS, but the relationship was not strong enough to conclude that functional appraisals are meaningfully associated with PTSS (de Haan et al., 2019). The other study that examined adaptive appraisals found that they mediated the relationship between child perceived social support and PTSS (Hitchcock et al., 2015).

#### Trauma Memory

Supplemental Table 3 summarizes the measures used and key findings for the 15 studies that examined trauma memory characteristics.

##### Measurement 

Studies used a variety of methods to assess trauma memories, including questionnaires that assess qualities of the memory and memory processing strategies, task-based measures, and coding schemes for quantifying memory qualities from the child’s trauma narrative. All studies used the child’s perceptions of the trauma memory, with no studies using parent-proxy report. Seven studies (20.6%) used the Trauma Memory Quality Questionnaire (TMQQ; Meiser-Stedman et al., 2007b) as a measure of the visual, sensory, and temporal aspects of the child’s memory for the traumatic event. Two studies used the Children’s Data-Driven Processing Questionnaire (CDDPQ; McKinnon et al., 2008) as a measure of peri-trauma processing, specifically how the child processed the physical/perceptual aspects of a trauma (i.e., “data-driven processing”) as opposed to processing the meaningful or contextual aspects of the trauma (i.e., “contextual processing”). One study used one Likert scale item to assess data-driven processing during the accident (Ehlers et al., 2003). Another study retrospectively identified items associated with trauma memory characteristics from a general trauma interview. Eight studies (23.5%) recorded the child’s retelling of the traumatic event as a trauma narrative and used a coding scheme to measure specific qualities of the trauma narrative. The types of memory qualities coded included types of processing (sensory, emotional, and thought processes; e.g., McKinnon et al., 2017b), the temporal organization of the narrative (e.g., Kenardy et al., 2007, Salmond et al., 2011), and the overall cohesion of the narrative (O’Kearney et al., 2007). Two studies used children’s trauma narratives and compared them to a witness report to assess the accuracy of the trauma memory (McKinnon et al., 2017a; Bray et al., 2018). Further, Bray and colleagues (2018) created a scenario in which they created a positive memory (gave each child a gift card while they were hospitalized), and then prompted the child to recall both the trauma memory and the positive memory two months later. Salmond and colleagues (2011) also prompted participants to recall another unpleasant memory not associated with the trauma (e.g., an argument with a friend). Each narrative was then coded for coherence, cohesion, and descriptive details. Lastly, Hitchcock and colleagues (2014) used a cued-recall task to assess the number of overgeneral memories related to positive and negative words.

##### Findings

Of the 15 studies that examined the role of trauma memory characteristics, nine (66.7%) found positive associations with PTSS, four (26.7%) found mixed effects based on the type of trauma memory characteristic, and two (13.3%) found no relationship. Overall, participants with higher TMQQ scores (i.e., poorer quality of memories, more intrusive memories, more sensory qualities related to memories) were more likely to meet criteria for ASD or PTSD and had higher PTSS. Importantly, Meiser-Stedman and colleagues (2007a) found that the TMQQ accounted for significant variance in PTSS not accounted for by PTSD intrusion symptoms. This highlights that the TMQQ measures a distinct construct not accounted for by the re-experiencing symptoms of PTSD (Meiser-Stedman et al., 2007a). TMQQ score was found to mediate the relationship between subjective life threat and meeting criteria for ASD (Meiser-Stedman et al., 2007b).

When assessing the overall quality and characteristics of children’s trauma narratives, there were mixed findings. Temporal disorganization within trauma narratives was consistently associated with higher PTSS (McKinnon et al., 2017b; Kenardy et al., 2007; Salmond et al., 2011; McGuire et al., 2021). The presence of more negative emotions within the narrative was significant for one study (McKinnon et al., 2017b), but not for another (Kenardy et al., 2007). More emotional content (McKinnon et al., 2017b), dissociative features (Kenardy et al., 2007), sensory qualities (McKinnon et al., 2017b; Salmond et al., 2011; O’Kearney et al., 2007; McGuire et al., 2021), and causal markers (e.g., “because”, “so”) were also found to be associated with PTSS. When comparing trauma narrative features to features of a positive event narrative (getting a gift card), Bray and colleagues (2018) found the opposite relationships to their hypotheses, such that children had similar accuracy, cohesion, and narrative detail in both narratives. Another study found that regardless of ASD diagnosis, trauma narratives had higher sensory content and lower positive emotion content than another negative event narrative (Salmond et al., 2011). When compared to a witness report of the accident, McKinnon and colleagues (2017a) found that memory accuracy did not predict PTSS.

Data-driven peri-trauma processing was found to predict later PTSS (McKinnon et al., 2008, 2017a). However, perceptions of memory quality were not found to mediate the relationship between data-driven processing and PTSS (McKinnon et al., 2008). Lastly, the number of overgeneral memories on a cued-recall task was found to be associated with early PTSS (at one- and three-months post-injury) but had the inverse relationship at six months (Hitchcock et al., 2014a, b).

#### Coping Strategies

For a summary of measurement and key findings for the 13 studies that measured coping strategies, see Supplemental Table 4.

##### Measurement

Twelve out of 13 studies used child self-report questionnaires. One study used a nurse report measure of the child’s use of dissociation as a coping strategy (the Child Stress Disorders Checklist; Saxe et al., 2003) while on an inpatient burn unit (Saxe et al., 2005). The most commonly used measure was the KidCope (*n =* 5), which asks children to rank how frequently they use a variety of adaptive (e.g., problem solving) and maladaptive coping strategies (e.g., social withdrawal, problem solving, self-criticism, wishful thinking), as well as the efficacy of each strategy (Spirito et al., 1988). Similarly, Marsac and colleagues (2017) used the How I Coped Under Pressure Scale (HICUPS; Ayers et al., 1996), which measures coping strategies within three domains: cognitive restructuring, social support, and avoidance coping. To measure specific cognitive coping strategies, two studies used the Children’s Response Style Questionnaire (CRSQ; Ziegert & Kistner, 2002) which examines the use of rumination and distraction for coping. Additionally, two studies used the White Bear Suppression Inventory (Muris et al., 1996), which measures the amount that an individual attempts to suppress intrusive thoughts. An additional two studies measured positive beliefs about worry as a coping strategy using responses on the Meta-Cognitions Questionnaire (MCQ; Cartwright-Hatton & Wells, 1997). Stallard and Smith (2007) and Ehlers and colleagues (2003) investigated cognitive coping strategies by developing items to assess rumination, thought suppression, distraction, and dissociation.

##### Findings

Eight studies (61.5%) found a significant relationship between coping strategy use and PTSS, with the additional four studies (30.8%) having mixed findings depending upon the type of coping strategy. Children with higher PTSS used more coping strategies overall than those with subthreshold symptoms (Marsac et al., 2014; Stallard et al., 2001).

Children with higher PTSS were more likely to use avoidant coping strategies (Marsac et al., 2017), including not going places they used to, although there was no association with avoiding accident stimuli specifically (Stallard, 2003). There were mixed findings about the association between PTSS and escape coping strategies. Marsac and colleagues (2016a) found no significant relationship, while other studies found significant relationships with use of distraction (Marsac et al., 2014; Stallard, 2003; Stallard et al., 2001; Stallard & Smith, 2007), social withdrawal (Marsac et al., 2014; Stallard, 2003; Marsac et al., 2013; Stallard et al., 2001), and resignation (Marsac et al., 2013). Persistent dissociation was conceptualized as a cognitive coping strategy for two studies. These studies found that dissociation as observed by the child’s nurse was associated with later PTSS (Saxe et al., 2005), as was self-reported dissociation (Ehlers et al., 2003).

Other cognitive coping strategies studied included thought suppression, rumination, and worry. Thought suppression was associated with increased PTSS when measured by the White Bear Suppression Inventory (Aaron et al., 1999; Vincken et al., 2012) or specific items developed by Ehlers and colleagues (2003) and Stallard and Smith (2007), but not when measured by the KidCope (Stallard, 2003). Several studies found that rumination was associated with PTSS (Stallard, 2003; Meiser-Stedman, 2009; Stallard & Smith, 2007; Meiser-Stedman et al., 2007a), but there was no relationship for Ehlers and colleagues (2003). Belief in worry as an effective coping strategy was associated with PTSS for one of two studies (Meiser-Stedman et al., 2007a). Blaming others for the accident was positively associated with PTSS (Marsac et al., 2014; Stallard, 2003).

In terms of active coping strategy use, Stallard and colleagues (2001) found a significant association with higher PTSS, although this was not replicated by Marsac and colleagues (2013). Cognitive restructuring (Marsac et al., 2017), emotion regulation (Stallard et al., 2001), and problem solving (Marsac et al., 2014) were each separately associated with having significant PTSS. Seeking social support was also positively associated with PTSS (Marsac et al., 2017).

## Discussion

The current review summarized 34 cross-sectional and longitudinal studies that assessed the relationship between psychological processing in the peri-trauma and recovery period and the development of PTSS. This literature represented studies from the last two decades in six countries that assessed psychological functioning following a variety of traumatic injuries. Overall, evidence suggested that PTSS is common following injury, although there is significant variability in which children develop clinically significant PTSS. In the studies included in this review, prevalence rates ranged from 3 to 38% and varied based on the time since injury and the cutoffs used (e.g., criteria for ASD or PTSD). The studies reviewed largely supported the applicability of the cognitive model by Ehlers and Clark (2000) to PTSS following pediatric injury, with variable results based on the construct studied.

Negative cognitive appraisals had the strongest support, with 100% of included articles finding a positive effect in predicting PTSS. Negative appraisals of the world (e.g., “anyone could hurt me”), psychological changes following an injury (e.g., “nothing good can happen to me anymore”), and guilt about the accident were strong predictors of PTSS up to six months post-injury. Although fewer studies examined functional or adaptive post-traumatic cognitive appraisals, there is evidence that these positive appraisals are a separate construct (de Haan et al., 2021) and that they are protective against the development of PTSS (Hitchcock et al., 2015). These findings highlight that a child’s *perception* of an event leading to injury is one of the most salient predictors of their psychological distress, which parallels prior literature demonstrating that objective injury factors are poor predictors of psychological outcomes (Trickey et al., 2012). Therefore, children with objectively milder injuries or short hospitalizations face risk for the development of PTSS, and the medical system should prioritize trauma-informed practices at all levels of acuity, including in the emergency department and outpatient clinics.

Trauma memory characteristics were also found to predict acute and chronic PTSS, although this relationship varied based on the methods used to assess memory. The current review found that children with clinically-significant PTSS had overall poorer memory quality when assessed using child self-report questionnaires (e.g., Meiser-Stedman et al., 2007a), although this relationship was mixed when assessing specific features of the child’s trauma narrative. Research with adults suggests that when individuals with PTSS attempt to intentionally recall the traumatic event, their intentional recall is fragmented, lacks contextual and temporal detail, and has intense sensory qualities that are experienced as if they are happening “here and now” (Ehlers & Clark, 2000). In the current review, temporal disorganization and more negative emotionality in the trauma narrative most consistently predicted higher PTSS (McKinnon et al., 2017b; Kenardy et al., 2007). However, one study found no significant relationships (Stallard, 2003).

Examining the qualities of a child’s trauma narrative has methodological challenges due to children’s ongoing cognitive development in areas such as memory processing, retrieval, and accuracy in time estimation (Pynoos et al., 1997). Further, children are still developing their expressive language skills and ability to describe non-routine events that have happened in the past (NIH, 2014). These factors may make a trauma narrative appear incomplete or lacking in context. Without well-validated coding schemes for children’s trauma memories, more research is needed to fine-tune the cognitive model of PTSD in the domain of trauma memories, to accurately assess risk factors for PTSS that are developmentally, linguistically, and cognitively appropriate for children. Within the current literature, using a child’s self-report of their memory qualities (e.g., “My memories of the frightening event are clear and detailed”) appears to be more closely associated with the development of PTSS.

Additionally, the potential impact of pain and medical treatment on memories of the traumatic event and hospitalization should be considered. Research suggests that there is a complex relationship between acute pain and PTSS. Hildenbrand and colleagues (2016, 2020) found that acute pain following pediatric injury significantly predicted PTSS at six months post-injury, even after controlling for other risk factors. Consistent with the fear conditioning model, which posits that peri-trauma pain triggers physiological stress responses that lead to over-consolidation of trauma memories and fear-conditioned responses (McLean et al., 2005), research suggests that pain indirectly predicts PTSS, with separation anxiety mediating the relationship (Saxe et al., 2005). There are mixed findings related to the influence of analgesic medications during the acute treatment phase on PTSS. Administration of opioid medications (e.g., morphine) during hospitalization may reduce PTSS risk (Morgan et al., 2003), although morphine use does not appear to mediate the relationship between pain and PTSS (Hildenbrand et al., 2020). To elucidate these relationships, future research should directly investigate the relationship between acute pain, medication treatment, psychological processing (particularly trauma memories), and PTSS.

The current review also identified several cognitive and behavioral coping strategies that are associated with PTSS. Overall, children with elevated PTSS used more coping strategies, including adaptive strategies (e.g., cognitive restructuring, seeking social support) as well as avoidant and escape coping (e.g., social withdrawal, distraction). This pattern suggests that children who are distressed following an injury may need to draw upon or attempt to use a greater repertoire of coping strategies (Stallard et al., 2001). Children with the most severe PTSS and overall distress may require formal intervention and coping support in order to benefit from these strategies. However, there is still little consensus on the most effective coping strategies to prevent or reduce PTSS, possibly due to the inter-relationships of coping style with other psychological processes.

Only one included study analyzed the relationships between the three major psychological processing constructs. Marsac and colleagues (2016) found that escape coping strategies mediated the relationship between appraisals and PTSS. Therefore, more negative appraisals regarding the psychological sequelae of the traumatic event (e.g., “I feel as if I am going crazy since the event”) lead to more attempts to cope using avoidant strategies, which then lead to PTSS. Further, the cognitive model highlights that behavioral and cognitive avoidance of triggers and rumination prevents individuals from changing appraisals, elaborating upon their trauma memory, and developing adaptive coping strategies to manage distress (Ehlers & Clark, 2000). These findings highlight the bidirectional, cyclical relationship between constructs. Because most of the current pediatric injury literature examines psychological processing variables separately, future research is needed to clarify the relationships among domains in the cognitive model. Given this initial evidence, models testing mediators and moderators of the relationship between psychological processing and PTSS, as well as structural equation modeling approaches (Marsac et al., 2016a, b) may more clearly elucidate these relationships.

The designs of current studies in this area hinder the ability to assess changes in PTSS or psychological processing over time. Firstly, many cross-sectional studies only assessed psychological processing and PTSS after time had passed following the injury. For example, Stallard and Smith (2007) collected data on coping strategies eight months post-injury. Therefore, it is possible that coping strategies occurred as a result and not as a precursor to PTSS. Further, another study (Morris et al., 2013) assessed cognitive appraisals and PTSS in the peri-trauma phase, several hours post-injury. Their cross-sectional findings highlight the relationship between appraisals and PTSS but are difficult to interpret with regards to future symptom trajectories. Assessment timing was also a barrier for some longitudinal study designs. For example, Nixon and colleagues (2010) assessed appraisals in the acute phase and PTSS in the chronic phase, but not vice versa. It is possible that some psychological processing variables are more impactful early in recovery from an injury (e.g., initial appraisals), whereas other factors (e.g., coping strategies) become more impactful over time. These limitations were reflected in the quality assessment ratings (particularly items 6, 7, and 10) and represent broader methodological challenges in the field. Future longitudinal research and meta-analyses are needed to tease apart these temporal relationships, particularly using a trajectories modeling approach.

Importantly, the majority of studies in the current review used child self-report of PTSS and psychological processing, given the internalizing nature of PTSS and the high rates of parent-child discrepancies (Scheeringa et al., 2006). Examining the role of parent psychological processing of the child’s traumatic event was beyond the scope of the current review. However, PTSS is common in parents of children with chronic illness, with a prevalence rate of roughly 20% (Cabizuca et al., 2009). Following injury, parent PTSS trajectories parallel child trajectories, with 78% of parents in the “resilient” trajectory, 8% in the “recovery” trajectory with acute symptoms that decline by six months, and with 14% of parents with “subclinical” chronic PTSS (Le Brocque et al., 2010). Given that the Ehlers and Clark (2000) model was developed with evidence from the adult literature, parental distress and adjustment and parental coping support are important factors to consider when examining psychological processing of trauma in children. Parents may influence child coping through coaching (e.g., direct instruction), modeling (e.g., the child observing the parent use particular strategies), and through general family functioning and emotion socialization (Miller et al., 1994). The role of parental cognitive appraisals of the injury is still unclear, but children with fewer negative cognitive appraisals were more likely to report clinically significant PTSS if their parents reported many negative appraisals (Morris et al., 2013). Therefore, there is initial evidence to support that parental PTSS and processing of the injury event should be added to the cognitive model; however, the directionality and strength of these relationships should be further studied.

### Limitations and Future Directions

The results of this review should be interpreted in light of some methodological limitations. First, the protocol of this review was not registered prospectively, which raises potential concerns for study bias and replication. Future systematic review protocols should be registered prospectively in order to avoid the introduction of bias in the review process. Only one reviewer conducted article screening and data extraction, which may have influenced the reliability of results and introduced bias. Due to the scope of the current literature in this area, only PTSS related to injury was examined. Price and colleagues’ (2016) model of medical traumatic stress was conceptualized using a transdiagnostic approach and includes medical trauma related to acute or life-threatening conditions, chronic illness, and injury. More knowledge related to the cognitive model of PTSD in children may be gleaned from combining pediatric medical populations in a larger review or meta-analysis. The current study also did not conduct any quantitative analysis or meta-analysis, limiting the conclusions that can be drawn about the role of psychological processing on PTSS. Lastly, because study publication dates ranged from 1999 to 2024, some studies used DSM-IV criteria for ASD and PTSD. There were significant changes to these diagnoses between the DSM-IV and DSM-5 (Pai et al., 2017). Because most studies used continuous symptom scores on validated measures as outcomes, results are unlikely to be significantly affected by this change, although this limitation should be considered when interpreting findings for older studies.

Quality assessment ratings identified several potential areas of bias in the current literature. The most common domain that led to “fair” quality was a lack of power or sample size justification. Lacking a sufficient sample size to reach statistical power may have prevented studies from detecting true differences, resulting in misleading findings and challenges with reproducibility. Further, only one study (Bray et al., 2018) reported an estimate of effect size or the magnitude of significant effects. Some studies used measures that were not yet validated, particularly for coding schemes to assess trauma narrative qualities. Other issues with study quality were related to the cross-sectional nature of over 40% of studies, including not measuring effects over multiple time points or allowing sufficient time for an effect to be present. Lastly, many studies did not report sample demographics, with the majority of studies not describing the racial/ethnic identities of their sample. Recent guidelines highlight the importance of comprehensive descriptions of study demographics using systems-centered and inclusive language to highlight systemic inequities and potential lack of generalizability for studies with predominantly White samples (Modi et al., 2024).

### Conclusions and Clinical Implications

The results of this review highlight the importance of trauma-informed practices across acute and follow-up medical care. Trauma-informed care recognizes the high prevalence of trauma and the need for universal procedures to limit the traumatic nature of medical care (Marsac et al., 2016a, b). Individuals on the “front lines” of the pediatric trauma systems, including physicians, nurses, and other health professionals providing acute care, should be knowledgeable about trauma-informed care practices and make changes to their care plans accordingly. Practices such as promoting physiological regulation through medications, relaxation strategies, and creative arts strategies are recommended in the acute care setting to reduce distress and re-traumatization (McDowell et al., 2022). Recognizing the impact of trauma on caregivers is also a vital component to trauma-informed care given the high rates of parental PTSS and the coping support that caregivers provide to their children. Studies show that emergency medicine physicians underestimate the likelihood that children will develop PTSS following injury (Ziegler et al., 2005). Although 96% of providers feel it is part of their job to decrease patient and family stress, less than a quarter reported implementing most trauma-informed medical practices (Cuneo et al., 2023). More education and training are needed to prevent PTSS in the acute phase. Further, hospital systems should increase screening of PTSS upon discharge from the inpatient setting, and define referral pathways for trauma-focused intervention. Several brief measures have been designed to screen for acute stress symptoms, with cutoff scores that indicate a need for follow-up (e.g., the Acute Stress Checklist; Kassam-Adams, 2006). Findings supporting the cognitive model may also lead to increased precision of measures to more accurately predict later PTSS. For example, assessing the child’s perceptions of the quality of their trauma memories may provide additional information on their risk for PTSS.

Future research on the cognitive model can help to fine-tune existing cognitive-behavioral interventions to be tailored to the trajectories of recovery and unique considerations for pediatric injury populations. Interventions that target positive reframing, adaptive coping strategies, and trauma narrative exposure may help to decrease the cycle of avoidance and distress about symptoms. Further, psychoeducation about stress reactions, the body’s stress response, and the relationship between thoughts, feelings, and behaviors can serve to normalize children and their families’ experiences, reduce negative appraisals, and promote resilience.

## Supplementary Information

Below is the link to the electronic supplementary material.

ESM 1 (DOCX 63.9 KB)

## Data Availability

Data is available upon request by contacting the corresponding author.
