# Supplementary material for: Applying the Cognitive Model of Post-Traumatic Stress to Examine the Role of Appraisals, Trauma Memory, and Coping Strategies Following Pediatric Injury: A Systematic Review
Source: J Child Adolesc Trauma. 2025 Feb 19;18(2):331–48. doi: 10.1007/s40653-025-00695-0 (PMC12129885; doi:10.1007/s40653-025-00695-0)
Supplement: Supplementary file 1 — (DOCX 63.9 KB) [file 40653_2025_695_MOESM1_ESM.docx]

**Supplemental Table 1**

*Study Demographics and Methods*

| **Authors, Year, Country** | ***N*** | **Child Demographics (age at injury, sex, race/ethnicity)** | **Injury Mechanisms/Types** | **Recruitment Setting** | **Study Design** | **Psychological Processing Variables Studied** |
| --- | --- | --- | --- | --- | --- | --- |
| Meiser-Stedman et al., 2007b  United Kingdom | 106^a^ | 11-16 years (M=24.0, SD=1.9)  63.2% male  Race/ethnicity not reported | Assault 56.6%  Road traffic accident 43.4% | Accident and Emergency department | Longitudinal | TM |
| McKinnon et al., 2017a  Australia | 57^b^ | 7-16 years (M=11.81, SD=2.13)  Sex not reported  Race/ethnicity not reported | Not reported | Two metropolitan hospitals | Longitudinal | TM |
| Hitchcock et al., 2015  Australia | 97 | 7-17 years (M=12.08, SD=2.80)  63% male  Race/ethnicity not reported | Road traffic accidents 47% Significant fall 12% Serious accidental injury 13% Other (assault, medical emergency, house fire) 27% | Recruited in-person at two metropolitan public hospital | Longitudinal | CA |
| Marsac et al., 2014  USA | 243 | 8-17 years (M=11.3, SD=2.5)  75% male  56% Black,  39% White | All road traffic accidents:  Hit by MV while riding a bike 50% Hit by MV while pedestrian 33% MV occupant involved in an MVC 16% Nearly 40% suffered an extremity fracture | In-hospital (ward or ICU) in a large, urban, Level I pediatric trauma center | Longitudinal | CS |
| Aaron et al., 1999  USA | 40 | 8-17 years (M=13.6, SD= 2.95)  47.5% male  85% White  15% African American | MVA 75%  Bicycle or ATV accident 10%  Hit by falling object 5%  Event leading to respiratory distress 5%  Physical assault other than child abuse 2.5%  Horse kick 2.5% | Following discharge from ICU or general pediatric units of two large teaching hospitals | Cross-sectional | CS |
| Stallard, 2003  United Kingdom | 97 | 7-18 years (M=14.62, SD=3.16)  53.6% male  Race/ethnicity not reported | All road traffic accidents:  Pedestrian 21.6% Passenger in car that crashed 59.8% On cycle/motorcycle that crashed 18.6%  Fractured bones 20.6%,  Blow to head 44.3% | Accident and Emergency department of a hospital over a one-year period | Cross-sectional | TM; CA; CS |
| Saxe et al., 2005  Australia | 72 | 7-17 years (M=11.20, SD=3.51) 66.7% male Race/ethnicity not reported | Acute burns | In-hospital at a specialized pediatric burn center | Longitudinal | CS |
| Kenardy et al., 2007  Australia | 87 | 7-15 years (M=11.20, SD=2.18)  67.8% male Race/ethnicity not reported | Not reported | Accident and Emergency centers at three hospitals | Longitudinal | TM |
| O'Kearney et al., 2007 Australia | 80 | 7-16 years (M=10.14, SD=2.28)  60% male  Race/ethnicity not reported | Fall 26.3% Bicycle/scooter/skateboard/rollerblade accident 16.3% Tripped over 15% Physical contact injury 11.3% Burn 8.8% Hit by car 6.3% Motorbike/go-cart accident 5% Animal injury 5% Cut/laceration 3.8% Hit by projectile 1.3% Near drowning 1.3% | Accident and Emergency centers at three hospitals | Cross-sectional | TM |
| McKinnon et al., 2008 Australia | 75 | 7-16 years (M=10.98, SD=2.40)  69% male  Caucasian 92% European 6% Other 1% | Serious sporting injuries 37% Road traffic accidents 7% Accidental injuries 52% Burns 4% | Emergency department or pediatric inpatient ward of two metropolitan hospitals | Cross-sectional | TM |
| Ellis et al., 2009 Australia | 97 | 7-17 years (M=12.08, SD=2.80)  63% male  Race/ethnicity not reported | Road traffic accident 47% Significant fall 12% Serious playground or sporting injury 13% Other 27% | Following presentation to the emergency department or admission to an inpatient ward | Cross-sectional | CA |
| Nixon et al., 2010  The Netherlands | 48 | 7-17 years (M=11.84, SD=2.67)  69% male  Caucasian 88% Other 12% | Road traffic accidents 60% Falls, assault, burns 23% Accidental injuries 17% | Emergency departments or pediatric inpatient ward of two metropolitan hospitals | Longitudinal | CA |
| Vincken et al., 2012  USA | 89 | 8-18 years (M=13.9, SD=2.8)  49.4% male  Race/ethnicity not reported | Road traffic accidents | Emergency room of two hospitals | Longitudinal | CS |
| Marsac et al., 2011  USA | 82 | 8-17 years  (M=12.1, SD=2.7)  70% male  African American 40% White 59% | Fractures 63.3% Sprains/strains 4.9% Contusions/lacerations 4.9% Head injuries 9.8% Organ injuries 7.3% Multiple traumas 3.7% Other 6.1% | Large, urban Level I pediatric trauma center | Longitudinal | CS |
| Morris et al., 2013  USA | 40 | 8-17 years (mean and SD not reported)  55% male Caucasian 82.5% African American 12.5% Other 5% | Traffic-related 25% Fall 27.5% Bike-related 12.5% Sports-related 12.5% Miscellaneous 22.5% | Emergency department | Cross-sectional | CA |
| Hitchcock et al., 2014  United Kingdom | 50 | 7-17 years (M=11.90, SD=3.31)  80% male  Caucasian 100% | Extreme sports accidents (Motocross or BMX); Incidents with dangerous equipment (power tools, spear fishing equipment); Falls (off a roof or cliff) Percentages not reported | Recruited in-person at a metropolitan public hospital | Longitudinal | TM; CS |
| McGuire et al., 2021  Australia | 126 | 6-13 years (M=9.8, SD=2.0)  62% male  Not reported ("predominantly Caucasian") | MVC 51.6% Serious falls or other serious accidental trauma 30.1% Acute medical episode 7.1% Assault 2.4% Other event (e.g., house fire, near drowning) 8.7% | Emergency department | Longitudinal | TM |
| McKinnon et al., 2017b  United Kingdom | 67 | 7-16 years (M=11.77, SD=2.13)  63% male  Caucasian 90% | Accidental injury (e.g., fall) 33% Serious sporting injuries (e.g., bike accident) 55% Road traffic collisions 8% Assaults 2% Burns 2% 28% minor injuries, 36% moderate injuries, 36% major injuries | Emergency department or pediatric ward | Longitudinal | TM |
| Meiser-Stedman et al., 2009 Switzerland & Germany | 59 | 10-16 years (M=14.0, SD=1.8)  54.2% male  Black (African) 10.2% Black (Caribbean) 15.3% Black (other) 16.9% Indian 1.7% Other 15.3% Caucasian 40.7% | Assault 52% Bicycle hit by MV 5.1% Passenger in MVA 8.5% Fall from bicycle 1.7% Motorbike passenger 1.7% Pedestrian hit by MV 30.5% | Consecutive admissions from the emergency department of one hospital | Longitudinal | TM; CS; CA |
| de Haan et al., 2019  United Kingdom | 114^c^ | 7-16 years (M=10.75, SD=2.55)  56.1% male  Race/ethnicity not reported | Road traffic accident or burn injury | Consecutive patients receiving inpatient or outpatient treatment at a children's hospital for the injury | Cross-sectional | CA |
| Meiser-Stedman et al., 2007a  United Kingdom | 93 | 10-16 years (M=13.9, SD=1.9)  64.5% male  Black (African) 16.1% Black (Caribbean) 17.2% Black (other) 22.6% Other 10.8% Caucasian 33.3% | Assault 55.9% Bicycle rider hit by MV 4.3% Fall from bicycle 1.1% Passenger in MVA 8.6% Motorbike rider 1.1% Motorbike passenger 1.1% Pedestrian 28.0% | Emergency department | Cross-sectional | TM; CA; CS |
| Stallard et al., 2001  United Kingdom | 97 | 5-18 years (M=14.62, SD=3.16)  53.6% male  Race/ethnicity not reported | All road traffic accidents:  Passenger in car accident 59.8% Pedestrian hit by vehicle 21.6% Motorcycle/cycle accident 18.6% | Accident and emergency department of one hospital | Longitudinal | CS |
| Seasons & Morrongiello, 2024  Canada | 103 | 14-17 years (M=15.8, SD=1.0)  78.6% male  White 58.3% Black 12.6% Asian 12.6% Indigenous Canadian 8.7% Latinx/Hispanic 1.9% | All skateboard injuries where medical treatment was received Strains/sprains 44.7% Fractures/breaks 35.9% Concussions 6.8% Cuts/bruises 2.9% Other injuries 9.7% | Flyers posted in local skate parks and community message boards, online forums and groups dedicated to skateboarding | Cross-sectional | CA |
| Stallard & Smith, 2007 Australia | 75 | 7-18 years (M=14.01, SD=3.36)  48.3% male  Race/ethnicity not reported | All road traffic accidents:  Pedestrian knocked over by vehicles 14.7% Passenger in MVC 64% Knocked off a cycle/motorcycle 21.3% | Accident and Emergency department at a hospital | Cross-sectional | TM; CA; CS |
| Bryant et al 2007 United Kingdom | 76 | 7-13 years (Males: M=10.50, SD=2.46; Females: M=8.77, SD=2.34)  65.8% male  Race/ethnicity not reported | Traumatic falls 47% MVC 7% Pedestrian or cyclist 30% Other injuries 16% | Consecutive child admissions to a major trauma hospital | Longitudinal | CA |
| Salmond et al., 2011 Australia | 50 | 8-17 years (M=13.50, SD=2.51)  50% male  ASD group: Caucasian 47.37% Black (African) 15.79% Black (Caribbean) 10.53% Black (other) 21.11% Other 0% Missing 5.26% Non-ASD group:  Caucasian 38.71% Black (African) 6.45% Black (Caribbean) 12.90% Black (other) 32.26% Other 9.68% Missing 0% | Assault 60% Road traffic accident 40% | Consecutive attendees at an Emergency Department | Cross-sectional | TM; CA |
| Salmon et al., 2007 Australia | 76 | 7-13 years (M=9.92, SD=2.55)  65.7% male  Race/ethnicity not reported | Traumatic falls 47% Motor vehicle passengers 7% Pedestrians or cyclists 30% Other injuries 16% | Consecutive admissions to a major trauma hospital, assessed at an outpatient clinic within 1 month of the injury | Cross-sectional | CA |
| Bray et al., 2018  United Kingdom | 38 | 7-17 years (M=12.4, SD=2.9)  63.2% male High PTSS: White 83.3%, European 0%, Aboriginal 0%;  Low PTSS: White 60.9%, European 13%, Aboriginal 4.3% | High PTSS:  Sporting accident 0%  Push bike accident 33.3%  Fall 25%  MVA 41.7%  Burns 0%  Laceration 0%  Other accident 0%  Low PTSS:  Sporting accident 13%  Push bike accident 17.4%  Fall 17.4%  MVA 17.4%  Burns 4.3%  Laceration 17.4%  Other accident 13% | Two metropolitan hospitals | Longitudinal | TM |
| Ehlers et al., 2003 USA | 86 | 5-16 years  (M=12.3, SD=2.86)  55% male  Race/ethnicity not reported | All road traffic accidents: Soft tissue injuries 73% Bony injuries 23% Evaluated in ED and determined to be uninjured 4% | Emergency department | Longitudinal | TM; CA; CS |
| Marsac et al., 2017 USA | 96 | 8-13 years (M=10.6, SD=1.7)  64.5% male  Black/African American 40.6% White 52.1% Other 7.3% Hispanic 4.2% Non-Hispanic 95.8% | Recreational activity  57.3% Sports 16.7% Motor-vehicle crash 15.6% Injured by animal 5.2% Gunshot wound 2.1% Kitchen incidents 3.1% 62.5% were fractures | Level 1 pediatric trauma center | Longitudinal | CA; CS |
| Marsac et al., 2016  USA | 688 | 8-17 years (M=11.8, SD=2.56)  72.5% male  43.1% Black 50.9% White | Traffic accident 37.6% Fall 24.3% Sports 34.7% Other 1.6% | Level 1 pediatric trauma center | 3 longitudinal studies (combined samples) | CA; CS |
| de Haan et al., 2020 Switzerland | 114 | 7-16 years (M=11.11, SD=2.40)  53.5% male Race/ethnicity not reported | Road traffic accident 71.9%  Burn injury 28.1% | Consecutive patients receiving inpatient or outpatient treatment at a children's hospital for the injury | Cross-sectional, measure validation study | CA |
| Haag et al., 2015  USA | 101 | 7-16 years (M=11.55, SD=2.70)  58.4% male  Race/ethnicity not reported | All road traffic accidents: Passenger in a car 15.8%  Motorcycle passenger 6.9% Pedestrian 33.7% Cyclist 27.7% Other 15.8% | Received inpatient or outpatient treatment for an injury | Baseline (cross-sectional) data from an RCT | CA |
| Hildenbrand et al., 2020  USA | 96 | 8-13 years (M=10.60, SD=1.71)  65% male  White: 52% Black/African American: 41% Hispanic: 4% Other: 3% | Recreational activities 57% Organized sports 17% Motor vehicle crash 16% Gunshot 2% Dog bite 5% Burn 3% 63% of injuries were fractures, 38% were another injury type | Recruited inpatient while hospitalized for an injury | Longitudinal | CA |

*Note.* TM= trauma memory; CA=cognitive appraisal; CS=coping strategies

^a^ Only Sample 2 was included in the review. Sample 1 consisted of children and adolescents recruited from two secondary schools and did not meet eligibility criteria.

^b^ Only Study 2 was included in the review. Study 1 included children who had undergone an orthopedic procedure and did not meet eligibility criteria.

^c^ Only the accidental trauma sample was included in the review. The interpersonal trauma sample did not meet eligibility criteria.

**Supplemental Table 2**

*Study Results for Cognitive Appraisals*

| **Author, Year** | ***N*** | **Data Collection Time Points** | **Measure of cognitive appraisals** | **Measure of PTSS** | **Summary of Findings** |
| --- | --- | --- | --- | --- | --- |
| Hitchcock et al., 2015 | 97 | T1: 1 month post-trauma T2: 6 months post-trauma | Negative appraisals: CPTCI Adaptive appraisals: AAQ, developed by the authors | CAPS child interview CPSS | Negative appraisals mediated the relationship between child-reported perceived social support and PTSS at T1; no relationship with parent-reported social support Adaptive appraisals mediated the relationship between child perceived social support and PTSS; no relationship with parent-reported social support Negative appraisals also mediated the impact of perceived social support on 6mo (T2) PTSS, but adaptive appraisals did not (no relationship with parent-reported social support) Negative appraisals at T1 significantly predicted T2 PTSS, although adaptive appraisals did not account for unique variance in T2 PTSS severity |
| Stallard, 2003 | 97 | Approximately 6 weeks post-accident | Appraisals of trauma sequelae: questions identified retrospectively from child interview (perception of enormous effect of injury, emotionally unrecovered, physically unrecovered) | CAPS child interview | Negative appraisals of trauma sequelae, feeling emotionally or physically unrecovered were correlated with PTSD |
| Ellis et al., 2009 | 97 | Within 4 weeks of trauma | CPTCI | ASC-Kids | CPTCI significantly correlated with PTSS (r=.69) No support for mediation model predicting that CPTCI mediated the relationship between social support and PTSS |
| Nixon et al., 2010 | 48 | T1: within 4 weeks of trauma  T2: 6 months post-trauma | CPTCI | CPSS | Maladaptive appraisals accounted for 30% of the variance in changes in PTSS from T1 to T2 and was associated with more PTSS overall |
| Morris et al., 2013 | 40 | A majority (95%) of children and their caregivers were recruited during their emergency department visit. The average length of time from injury to assessment in the ED was 2.95 hr (SD 5.55) | Measure created by Stallard and Smith (2007), early version of the CPTCI | RIES-C | Significant correlations between child appraisal of stressor severity, child appraisal of permanent change, child appraisal of heightened future danger, and child appraisal of alienation from other people  Significant main effects for child appraisal of stressor severity, child appraisal of permanent change, child appraisal of heightened future danger, and child appraisal of alienation when predicting child PTSS Significant interaction between parent and child appraisals of permanent change in predicting child PTSS Significant interaction between parent and child appraisals of future danger in predicting child PTSS High permanent change appraisals in parents did not appear to impact child PTSS for children who also endorsed high appraisals of permanent change. However, children who endorsed low levels of permanent change reported experiencing elevated levels of PTSS if their parents endorsed high levels of permanent change Children with low levels of future danger appraisals, whose parent endorsed high levels of future danger, reported elevated PTSS |
| Meiser-Stedman, 2009 | 59 | T1: 2-4 weeks post-injury T2: 6 months post-injury | CPTCI | ADIS child interview to assess for PTSD  CRIES | Children with PTSD at T2 had more maladaptive post-traumatic appraisals  Maladaptive appraisals assessed at 6 months on both subscales of the CPTCI were significantly and independently associated with 6-month symptom levels/diagnosis even after controlling for 4-week symptom levels/diagnosis Examination of each CPTCI subscale separately revealed that scores on the Permanent and Disturbing Change subscale significantly mediated the relationship between T1 and T2 PTSS. The mediation effect of the Fragile Person in a Scary World subscale was nonsignificant |
| deHaan et al., 2019 | 114 | 3 months post-injury | CPTCI | RI | Younger age was associated with more dysfunctional post-traumatic appraisals  Child dysfunctional post-traumatic appraisals mediated the relationship between younger age and lower parental educational level on child PTSS. Child dysfunctional post-traumatic appraisals positively predicted child PTSS |
| Seasons & Morrongiello, 2024 | 103 | Not reported | Vulnerability appraisals: How likely did you think it was that you would get hurt? (prior to the injury), on a Likert scale; Attributions to bad luck: Probed for how much the adolescent believed the cause for injury was due to bad luck (0-100%) | CATS symptom checklist option. Participants were asked to retrospectively report on the symptoms they experienced in the 2 weeks following the most severe skateboarding injury they experienced in the past year that required being seen by a medical professional. | No significant statistical direct effects were found for injury appraisals of perceived vulnerability and attributions of injury to bad luck on PTSS Attributions to bad luck appeared to negatively moderate the relationship between pain at injury and PTSS, such that as participants’ beliefs about the injury being due to bad luck increased, the relationship between pain and PTSS became weaker |
| Bryant et al., 2007 | 76 | T1: Within 4 weeks of admission  T2: 6 months post-admission | CPTCI | T1: CASRQ (interview with a Masters-level clinical psychologist) T2: RI (interview with a clinical psychologist who was unaware of the patient's diagnostic status at the acute phase) | The CPTCI- Feeble subscale accounted for 13% of the variance in PTSD symptoms when accounting for age and injury severity score  The CPTCI- Change subscale did not significantly contribute to the prediction of PTSD In the final model, only CPTCI-Feeble subscale significantly predicted chronic PTSS |
| Salmond et al., 2011 | 50 | Within 2-4 weeks post-injury | CPTCI | CPSS ADIS-C to identify diagnosis of ASD | Children with ASD had higher cognitive misappraisals of the trauma on the CPTCI compared to non-ASD controls |
| Salmon et al., 2007 | 76 | Within 4 weeks of trauma | CPTCI | CASRQ | Negative appraisals accounted for 44% of the variance in their acute stress reactions.  In particular, the Feeble scale accounted for nearly half of the variance and was the strongest predictor of acute stress symptoms Children with ASD scored higher on both CPTCI sub scales (Feeble and Change) |
| Ehlers et al., 2003 | 86 | T1: 2 weeks post-trauma  T2: 3 months post-trauma T3: 6 months post-trauma | Three questions on a Likert scale about (1) Negative interpretation of intrusive memories, (2) Alienation from other people, and (3), Appraisals related to unfairness | Severity of PTSD symptoms as defined by DSM-IV Used items from the IES and the RI Authors constructed a new item whenever one was needed to capture a DSM-IV symptom | Measures of negative cognitive appraisal correlated with PTSS at 3 months and 6 months A hierarchical regression model predicting PTSS predicted an additional 35.6% of the variance in PTSS when adding appraisals and cognitive strategy use at 2 weeks post-injury Another regression model found that cognitive variables at 3 months significantly improved the prediction of PTSD severity at 6 months, explaining 38.5% of the variance (together with sex and stressor severity explained 53% of the variance) |
| Marsac et al., 2016 | 688 | T1: within 4 weeks of injury T2: 6-12 weeks post-injury T3: 6 months post-injury | 2 questions measuring threat appraisals ("Did you feel really afraid?") and subjective life threat ("Did you think you might die?") | Dichotomous outcomes (elevated PTSS or not); Study 1: CASRQ at T1, CAPS interview for T3 Studies 2 and 3: CPSS | In structural equation models, threat appraisals related to the event were concurrently related to PTSS status at baseline Escape coping (distraction, social withdrawal, self-criticism, wishful thinking, resignation) predicted PTSS status and mediated the relationship between appraisals and T3 PTSS status |
| deHaan et al., 2020 | 114 | Between 6 and 43 days post-accident (M = 18.08 days, SD = 7.5) | FPTCQ CPTCI | RI | Negative correlation between PTSS and FPTCQ (-.35), but not enough relationship to make a strong case that functional PTCs are meaningfully associated with PTSS Stronger association with functional PTCs by age, with a significant stronger association for adolescents compared to children Significant positive correlation between PTSS and CPTCI (.67) FPTCQ and CPTCI were moderately negatively correlated (-.44), indicating that the FPTCQ expands upon the concept of dysfunctional PTCs |
| Haag et al., 2015 | 101 | Approximately 10 days post-accident | Guilt appraisals measured with one question: "Do you feel guilty for causing the accident?" with a Likert scale response | IBS-A-KJ, a standardized clinical interview based on the DSM-IV criteria | Guilt appraisal significantly predicted ASD total severity when controlling for sex, age, SES, injury severity, inpatient treatment, pre-trauma psychopathology, and maternal PTSS Guilt appraisal predicted intrusion and avoidance symptoms Guilt was a marginally significant predictor for dissociation symptoms Guilt was not significantly associated with hyperarousal symptoms |
| Meiser-Stedman et al., 2007a | 93 | Few weeks post-injury | Survey questions about appraisals of subjective severity of threat, anger towards other person, anger/guilt towards self | ADIS-C  child and parent versions RIES-C | Subjective threat appraisal at the time of the trauma was significantly correlated with ASD and early PTSD Subjective threat appraisal significantly predicted meeting criteria for ASD ASD dissociation symptoms were significantly associated with subjective appraisal of threat  Anger towards others was not associated with scores on the RIES-C or receiving an ASD or PTSD diagnosis  Anger towards self was significantly correlated with RIES-C score (r=.26) |
| Marsac et al., 2017 | 96 | T1: within 2 weeks post-injury T2: 6-week follow-up T3: 12-week follow-up | Global appraisals: CPTCI Trauma-specific appraisals of injury event: ASC-Kids | CPSS | Significant relationship between each type of appraisals and PTSS at every time point Global appraisals and trauma-specific appraisals significantly predicted T1 PTSS but not T3 PTSS |
| Hildenbrand et al., 2020 | 96 | T1: within 2 weeks post-injury  T2: 12 weeks post-injury | 4 items on the ASC-Kids to assess appraisals of the injury event | CPSS | Traumatic appraisals significantly correlated with T1 and T2 PTSS. Structural equation modeling predicting PTSS found that traumatic appraisals of the injury event was a significant predictor of PTSS at T1 but not at T2. |

*Note.* CAPS=Clinician-Administered PTSD Scale for Children and Adolescents; CPSS= Child Post-Traumatic Stress Scale; ASC-Kids= Acute Stress Checklist for Children; RIES-C=Impact of Event Scale- Revised; ADIS-C= Anxiety Disorders Interview Schedule for the DSM-IV: Child Version; RI= UCLA PTSD Reaction Index; CATS=Child and Adolescent Trauma Screen; CASRQ=Child Acute Stress Reaction Questionnaire; IBS-A-KJ= Interview zur Akuten Belastungsstorung bei Kindern und Jugendlichen; CPTCI= Child Post-Traumatic Cognitions Inventory; AAQ=Adaptive Appraisals Questionnaire; FPTCQ=Functional Posttraumatic Cognitions Questionnaire

**Supplemental Table 3**

*Study Results for Trauma Memory Characteristics*

| **Author, Year** | ***N*** | **Data Collection Time Points** | **Measure of trauma memory** | **Measure of PTSS** | **Summary of Findings** |
| --- | --- | --- | --- | --- | --- |
| Meiser-Stedman et al., 2007b | 106 | T1: 2-4 weeks post-injury  T2: 3 months post-injury  T3: 6 months post-injury | TMQQ | RIES-C  ADIS-C: Child and Parent Versions | Participants with ASD scored significantly higher on the TMQQ than participants without ASD Participants with PTSD scored significantly higher on the TMQQ than participants without PTSD TMQQ positively correlated with PTSS on the RIES-C The TMQQ accounted for 2.4% of the variance not associated with the RIES-C intrusion subscale, and the RIES-C intrusion sub-scale accounted for 19.1% of the variance not associated with the TMQQ, indicating that the TMQQ is not simply an index of re-experiencing symptoms of PTSS |
| McKinnon et al., 2017a | 57 | T1: within 1 month post-injury T2: 3 months post-injury | Memory interview: 17 probed recall questions to ask about the prototypical features of the accident (e.g., people present, time of accident, what happened afterwards); also endorsed how confident they felt in their memory of the trauma Accuracy was compared to witness reports (in most cases parents); CDDPQ | CPSS | T1 data-driven processing and peri-event fear predicted T2 PTSS T1 self-reported fear and T1 data-driven processing did not predict memory accuracy or confidence at T2 T1 memory accuracy and T1 confidence did not predict T2 PTSS In a hierarchical regression, when controlling for age, data-driven processing and fear accounted for 20.5% of the variance in PTSS In the final model, only data-driven processing was a significant predictor |
| Stallard, 2003 | 97 | Approximately 6 weeks post-accident | Questions identified retrospectively from child interview (confusion during trauma, incomplete memory during accident) | CAPS (interview format) | Trauma memory characteristics (feeling confused, unable to remember parts of the accident) were not associated with PTSD |
| Kenardy et al., 2007 | 87 | T1: 4-7 weeks post-trauma T2: 6 months post-trauma | Child was asked to give a verbal narrative of their injury to an interviewer Narrative was recorded and coded for specific themes of dissociation, emotion, temporal disorganization, and dissociative amnesia Also counted number of positive emotion and negative emotion, non-classifiable emotion, or absence of emotion Temporal disorganization was coded as a yes/no if the narrative made no logical sense, or the child repeated aspects of the accident Dissociative amnesia coded if child could not remember aspects of the trauma Dissociative symptom was coded if they met criteria for one of the following: absence of emotion, temporal disorganization, or dissociative amnesia | ADIS-C (PTSD category) | 47.1% of children had dissociation in their trauma narratives The narrative themes of absence of emotion and dissociative amnesia were not significantly related to parent-reported individual symptoms of hyperarousal, avoidance or re-experiencing, nor subsyndromal PTSD, at T1  Children who reported at least one symptom of dissociation during the narrative did not differ significantly from those who did not in terms of concurrent, parent-reported subsyndromal PTSD or individual symptoms of PTSD  Children who expressed temporal disorganization during narratives were more likely to be reported as showing one or more moderate to severe symptoms of hyperarousal, avoidance and/or re-experiencing at T1 compared to children who did not demonstrate temporal disorganization in the narratives At T2, children who reported at least one symptom of dissociation during the narrative were significantly more likely than children who reported no dissociative symptoms to experience hyperarousal as reported by their parent  There was also a significant association between absence of emotion in the narrative and parent-reported hyperarousal at 6 months post-accident |
| McKinnon et al., 2008 | 75 | Within 4 weeks post-injury | CDDPQ TMQQ | ASC-Kids | Data-driven processing was significantly correlated with the acute stress sub scales of the ASC-Kids and TMQQ Perceptions of memory quality mediated the relationship between data-driven processing and intrusion PTSS Perceptions of memory quality did not mediate the relationship between data-driven processing and arousal PTSS Perceptions of memory quality mediated the relationship between peritraumatic fear and intrusion PTSS |
| Hitchcock et al., 2014 | 50 | T1: 1 month  T2: 3 months T3: 6 months post-trauma | Autobiographical Memory Test, a cued-recall task (number of overgeneral memories on the task) | CPSS | Time since trauma exposure moderated the relationship between overgeneral memory and PTSS: there was a positive relationship between overgeneral memory and PTSS at T1 and T2, but there was a significant negative relationship at T3 |
| McKinnon et al., 2017b | 67 | T1: within 4 weeks of injury T2: 8 weeks later | Trauma narrative with previously validated coding scheme Counts were used to determine the number of lexical (sensory, emotional, thought processing words) and cohesive (additive, temporal, causal, comparative) devices used in each in narrative | T1: CASQ T2: CPSS | Sensory elements were most commonly seen in narratives, followed by emotion references, thought processes, and then references to impaired cognitive processing (e.g., "I couldn't think") The use of negative emotions and the temporal index (organization of the narrative temporally) were correlated with ASD symptoms at T1 Narrative features and perceptions of trauma memory quality accounted for 10.2% and 12.5% of variance in ASD symptoms; narrative features no longer significant when accounting for perceptions of trauma memory quality Children with probable ASD included significantly more negative emotions, had a lower temporal organization score, and had significantly higher scores on the TMQQ Children with probable PTSD at T2 had lower temporal index score at T1, included significantly more emotions, and had significantly higher scores on the TMQQ A reduction in perceptions of trauma memory quality (i.e., the less fragmented and sensory in nature they became) predicted a reduction PTS symptoms over time |
| Meiser-Stedman, 2009 | 59 | T1: 2-4 weeks post-injury T2: 6 months post-injury | TMQQ | ADIS-C interview; RIES-C | Children with PTSD at T2 had poorer memory quality on the TMQQ |
| Stallard & Smith, 2007 | 75 | About 8 months post-injury | Trauma memory: assessed by 2 items (confusion during accident and difficulty remembering parts of the accident) | CAPS interview;  RIES-C | Model with all cognitive predictors predicting PTSS accounted for 64% of the variance (using the CAPS-C as the outcome) When using the IES-8 as the outcome, still significant and accounted for 61% of the outcome. The item "feeling confused/muddled during the accident" did not add predictive power and was excluded from the final model |
| Salmond et al., 2011 | 50 | Within 2-4 weeks post-injury | Verbal narrative of trauma memory and another negative event memory prior to the trauma (e.g., an argument with a friend) TMQQ | CPSS;  ADIS-C interview | Those with ASD had significantly elevated levels of disorganization in their trauma narrative compared to children without ASD and when compared to their narrative of a non-traumatic negative event Regardless of ASD diagnostic status, trauma narratives had significantly higher sensory content and significantly lower positive emotion content compared to the unpleasant comparative narrative  Acute symptom severity was significantly predicted by the level of disorganization in the trauma narrative ASD group scored higher on the TMQQ, indicating their memories are more sensory and fragmented than those in the non-ASD group for both the trauma and the other negative event narrative |
| Bray et al., 2018 | 38 | Approached inpatient and given gift card in a standardized manner; Follow-up assessment via phone 1 week post-injury (T1) and 2 months post-injury (T2) | TMQQ  Cued recall task to assess memory accuracy for the injury (negative event) and for receiving a gift card (positive event). Child’s memory compared to witness recall of events. Both narratives were coded by researchers for coherence, cohesion, and descriptive details using a standardized protocol | T1: ASC-Kids  T2: CPSS | There was no significant difference between narrative details for the negative or positive event between high and low PTSS groups, trending towards high PTSS group having more detail for the negative event than the positive event (d=.50)  High PTSS group reported more detail in their narratives at T2 than at T1 for both events (d=.93), while low PTSS showed little change (d=.002) Children had a high level of accuracy for both negative and positive events For cohesion of memories, high PTSS group did not have differences between the negative or positive event; trended towards more cohesion for negative event than positive event (d=.40 and .13, respectively) Children in both groups were more confident in their memories for the negative event than the positive event (high PTSS: d=0.79, low PTSS: d=1.15) |
| Ehlers et al., 2003 | 86 | T1: 2 weeks  T2: 3 months and 6 months | Data driven processing: One question about whether they were muddled/confused during the accident on a Likert scale  *All questions developed by the authors | Severity of PTSD symptoms as defined by DSM-IV Used items from the RIES-C and the RI Authors constructed a new item whenever one was needed to capture a DSM-IV symptom | Data driven processing was correlated with PTSS at 3 months and trended towards significance at 6 months Regression model found that cognitive variables together at 3 months significantly improved the prediction of PTSD severity at 6 months, explaining 38.5% of the variance (together with sex and stressor severity explained 53% of the variance) |
| O’Kearney et al., 2007 | 80 | 4 to 7 weeks post-injury | Child was asked to give a verbal narrative of their memory of the accident Narratives were coded for: (1) measures of the distribution of lexical categories (emotional, conceptual and sensory/perceptual); (2) indices of narrative connectedness (cohesion) and (3) indices of narrative organization (coherence) | RIES-C | There was a significant moderate association between higher sensory/perceptual references (e.g. saw, looked, felt) and lower levels of intrusion symptoms  Children’s use of more causal cohesive markers (e.g. because, so, therefore) in their memory narrative was also associated with higher levels of intrusion symptoms, but was not related to avoidance Children with clinically significant PTSS used fewer negated conceptual references than children in the rest of the sample Lower age, fewer sensory/perceptual references and more causal cohesive markers predicted higher intrusion scores for the high symptomatic subgroup of children |
| McGuire et al., 2021 | 126 | T1: 1 month post-injury T2: 6 months post-injury | ATMQQ Children provided a verbal narrative of the traumatic event. Narrative was coded according to standardized procedures for repetition, organized thoughts, disorganized thoughts, negative feelings, pain utterances, and sensory utterances; a global coherence rating was also rated | RI (child-report) ADIS-C, parent and child report | Longer trauma narratives associated with higher PTSS at baseline Both sensory and disorganization subscales of the ATMQQ were associated with PTSS at both time points None of the trauma narrative indices were associated with child PTSS at either time point After controlling for age, trauma memory characteristics on the ATMQQ significantly predicted 48.5% of variance in T1 PTSS, with both self-reported disorganization and sensory qualities of memories independently associated with concurrent PTSS After controlling for age, initial trauma memory characteristics predicted 15% of variance in 6-month PTSS, with both self-reported disorganization and sensory qualities of memories as significant independent predictors. However, after controlling for age and initial symptoms, the self-reported trauma memory characteristics of disorganization and sensory qualities no longer predicted additional variance in 6-month PTSS When using the PTSS severity score from the ADIS interview (which was a combination of parent and child report), results were broadly similar |
| Meiser-Stedman et al., 2007a | 93 | Few weeks post-injury | TMQQ | ADIS-C interview, RIES-C | More sensory memories on the TMQQ was significantly correlated with ASD TMQQ score significantly predicted meeting criteria for ASD TMQQ mediated the relationship between subjective threat and meeting criteria for ASD Patterns of correlations for meeting criteria for ASD mimicked pattern for early PTSD criteria |

*Note.* RIES-C=Impact of Event Scale- Revised; ADIS-C= Anxiety Disorders Interview Schedule for the DSM-IV; TMQQ=Trauma Memory Quality Questionnaire; CDDPQ=Children's Data-Driven Processing Questionnaire (CDDPQ); CPSS= Child Post-Traumatic Stress Scale; CAPS=Clinician-Administered PTSD Scale for Children and Adolescents; ASC-Kids= Acute Stress Checklist for Children; CASQ= Child Acute Stress Questionnaire; ATMQQ= Adapted Trauma Memory Quality Questionnaire

**Supplemental Table 4**

*Study Results for Coping Strategies*

| **Author, Year** | ***N*** | **Data Collection Time Points** | **Measure of coping strategies** | **Measure of PTSS** | **Summary of Findings** |
| --- | --- | --- | --- | --- | --- |
| Marsac et al., 2014 | 243 | T1: 1 month post-injury T2: 6 months post-injury | KidCope | CASQ | Children most often reported using wishful thinking and cognitive restructuring strategies Children with significant acute stress reactions were more likely to later report using distraction, social withdrawal, problem-solving, and blaming others as coping strategies Acute stress reaction severity was positively associated with the total number of coping strategies they used at T2 |
| Aaron et al., 1999 | 40 | Approximately 1 month post-injury | WBSI | RIES-C | Thought suppression was correlated with scores on the RI and the IES.  RI and IES total scores were significantly higher for those who were "high suppressors" than "low suppressors" There was a significant main effect of thought suppression on PTSS |
| Stallard, 2003 | 97 | Approximately 6 weeks post-accident | KidCope | CAPS, interview format | Behavioral avoidance, not going places, being less sociable, social withdrawal were associated with PTSD Maintaining cognitive strategies of distraction and rumination were associated with PTSD Avoiding accident stimuli not associated with PTSD Thought suppression (trying to forget about accident) not associated with PTSD |
| Saxe et al., 2005 | 72 | T1: In-hospital (about 10 days post-admission)  T2: 3 months later | Numbing and dissociation scale of the CSDC (nurse report of dissociation symptoms) | RI | Dissociation symptoms and PTSS were significantly correlated Dissociation mediated the relationship between total burn area and PTSS Both anxiety and dissociation independently contributed to the risk for PTSD |
| Vincken et al., 2012 | 89 | T1: within 2 weeks post-injury T2: 2 months post-injury | WBSI | CPSS | WBSI scores at two weeks were strongly associated with total PTSS at 2 weeks, and with the three symptom clusters of PTSS WBSI scores were also significantly related to PTSS at T2 Correlations between WBSI scores and PTSS at 2 months were not significant after controlling for PTSS at 2 weeks |
| Marsac et al., 2011 | 82 | T1: 2 weeks post-injury T2: 3 months post-injury | KidCope | CPSS | Frequently reported coping strategies included wishful thinking, seeking social support, distraction, and cognitive restructuring Children who went on to develop PTSS were more likely to have utilized avoidant (social withdrawal) or negative (resignation) coping strategies in the 2 weeks post-injury Significant PTSS at 3 months was associated with the use of social withdrawal at both time points, but was related to the use of resignation only at 2 weeks and not at 3 months No statistically significant relationship was found between active coping behaviors post-injury and (T1, T2) and PTSS outcome (T2) |
| Meiser-Stedman, 2009 | 59 | T1: 2-4 weeks post-injury T2: 6 months post-injury | CRSQ, rumination subscale  MCQ, positive beliefs about worry subscale | ADIS-C, interview  RIES-C | Children with PTSD at T2 had more rumination  There were no significant differences between those with and without PTSD on positive beliefs about worry as a coping strategy |
| Stallard et al., 2001 | 97 | T1: 6 weeks post-injury T2: 8 months post-injury | KidCope | CAPS, interview format | Almost all children reported using wishful thinking, followed by cognitive restructuring and social support as next most frequent Almost all coping strategies (including both approach/problem-focused and avoidance/emotion-focused) were rated as effective Self-criticism was used least often and was viewed as least effective Children with PTSD at T1 were more likely to report using avoidant/emotion-focused strategies of social withdrawal, distraction, emotional regulation, and blaming others. No other significant relationships Children with PTSD at T2 used more strategies overall than those without PTSD The coping strategies of blaming others and social withdrawal had the best predictive power when predicting PTSD diagnosis at T1 Children with PTSD at T2 used more distraction and social withdrawal at T1 Children with PTSD at T2 used more coping strategies at T1 Differences in coping strategy use between T1 and T2 for: self-criticism, problem-solving, emotional regulation, and social support |
| Stallard & Smith, 2007 | 75 | About 8 months post-injury | Cognitive coping strategies: Rumination (4 items), thought suppression (2 items), distraction (2 items) | CAPS, interview format  RIES-C | All coping measures were correlated with PTSS  Created a factor related to coping style (rumination, suppression, and distraction); full model predicting PTSS with all cognitive variables accounted for 64% of the variance (using the CAPS-C as the outcome) When using the IES-8 as the outcome, still significant and accounted for 61% of the outcome |
| Ehlers et al., 2003 | 86 | T1: 2 weeks  T2: 3 months T3: 6 months post-injury | Cognitive strategies: Likert scale items related to rumination, thought suppression, and persistent dissociation  *All questions developed by the authors | Severity of PTSD symptoms as defined by DSM-IV Used items from the RIES-C and the RI Authors constructed a new item whenever one was needed to capture a DSM-IV symptom | Maintaining cognitive strategies correlated with PTSS at 3 months and 6 months  Rumination was approaching significance at 6 months A hierarchical regression model predicting PTSS predicted an additional 35.6% of the variance in PTSS when adding appraisals and cognitive strategy use at 2 weeks post-injury Another regression model found that cognitive variables at 3 months significantly improved the prediction of PTSD severity at 6 mo, explaining 38.5% of the variance (together with sex and stressor severity explained 53% of the variance) |
| Marsac et al., 2016 | 688 | T1: within 4 weeks of injury T2: 6-12 weeks post-injury T3: 6 months post-injury | KidCope | Due to each study using different measures, used dichotomous scores (clinically significant PTSS or not) Study 1: CASQ at T1, CAPS for T3 Studies 2 and 3: CPSS | Escape coping (distraction, social withdrawal, self-criticism, wishful thinking, resignation) predicted PTSS status and mediated the relationship between appraisals and T3 PTSS status Control coping (cognitive restructuring, problem-solving, social support, relaxation) was not associated with PTSS over time |
| Meiser-Stedman et al., 2007a | 93 | Few weeks post-injury | CRSQ-rumination subscale MCQ, positive beliefs about worry subscale | ADIS-C, child and parent versions RIES-C | Ruminative responses to negative affect on the CRSQ, and positive endorsement of worry on the MCQ were all significantly correlated with ASD A combination of rumination, positive beliefs about worry, and anxiety sensitivity predicted ASD Patterns of correlations for meeting criteria for ASD mimicked pattern for early PTSD criteria ASD dissociation symptoms were significantly associated with positive beliefs about worry and subjective appraisal of threat, trending significant with rumination |
| Marsac et al., 2017 | 96 | T1: within 2 weeks post-injury T2: 6-week follow-up T3: 12-week follow-up | HICUPS | CPSS | Significant correlations between each type of coping and PTSS at each time point  In structural models, significant associations emerged between T1 PTSS and each type of T2 Coping, both in individual models and combined models. Only Avoidance Coping was in turn predictive of T3 PTSS in the individual models |

CASQ= Child Acute Stress Questionnaire; RIES-C=Impact of Event Scale- Revised; CAPS=Clinician-Administered PTSD Scale for Children and Adolescents; RI= UCLA PTSD Reaction Index; CSDC=Child Stress Disorders Checklist; WBSI=White Bear Suppression Inventory, CPSS= Child Post-Traumatic Stress Scale; CRSQ=Children's Response Style Questionnaire; MCQ= Meta-Cognitions Questionnaire; ADIS-C= Anxiety Disorders Interview Schedule for the DSM-IV; HICUPS=How I Coped Under Pressure Scale
